# A Modified Calculation Improves the Accuracy of Predicted Postoperative Lung Function Values in Lung Cancer Patients

**DOI:** 10.1007/s00408-021-00464-4

**Published:** 2021-08-13

**Authors:** G. Schlachtenberger, F. Doerr, H. Menghesha, L. Hagmeyer, T. Leschczyk, C. Gaisendrees, M. Michel, T. Wahlers, K. Hekmat, M. B. Heldwein

**Affiliations:** 1 Department of Cardiothoracic Surgery, University Hospital of Cologne, Kerpenerstrasse 62, 50937 Cologne, Germany; 2 Clinic for Pneumology and Allergology, Hospital Bethanien, Aufderhöher Strasse. 169-175, 42699 Solingen, Germany; 3 School of Medicine, University of Cologne, Cologne, Germany; 4 Faculty of Mathematics and Natural Sciences, Institute of Zoology, University of Cologne, Zuelpicher Strasse 47b, 62, 50674 Cologne, Germany

**Keywords:** Customizing the ppoFEV1, ppoFEV1, Non-small cell lung cancer, Lung function, Risk stratification

## Abstract

**Purpose:**

Preoperative pulmonary function testing is mandatory for non-small cell lung cancer (NSCLC) surgery. The predicted postoperative FEV1 (ppoFEV1) is used for further risk stratification. We compared the ppoFEV1 with the postoperative FEV1 (postFEV1) in order to improve the calculation of the ppoFEV1.

**Methods:**

87 patients voluntarily received an FEV1 assessment 1 year after surgery. ppoFEV1 was calculated according to the Brunelli calculation. Baseline characteristics and surgical procedure were compared in a uni- and multivariate analysis between different accuracy levels of the ppoFEV1. Parameters which remained significant in the multinominal regression analysis were evaluated for a modification of the ppoFEV1 calculation.

**Results:**

Independent factors for a more inaccurate ppoFEV1 were preoperative active smoking (odds ratio (OR) 4.1, confidence interval (CI) 3.6–6.41; *p* = 0.01), packyears (OR 4.1, CI 3.6–6.41; *p* = 0.008), younger age (OR 1.1, CI 1.01–1.12; *p* = 0.03), and patients undergoing pneumectomy (OR 5.55, CI 1.35–23.6; *p* = 0.01). For the customized ppoFEV1 we excluded pneumonectomies. For patients < 60 years, an additional lung segment was added to the calculation. ppoFEV1 = preFEV1 × $1-\left(\frac{\text{Lung segments resected} + 1}{\text{Total number of segments}}\right)$. For actively smoking patients with more than 30 packyears we subtracted one lung segment from the calculation ppoFEV1 = PreFEV1 × $1-\left(\frac{\text{Lung segments resected} - 1}{\text{Total number of segments}}\right)$.

**Conclusion:**

We were able to enhance the predictability of the ppoFEV1 with modifications. The modified ppoFEV1 (1.828 l ± 0.479 l) closely approximates the postFEV1 of 1.823 l ± 0.476 l, (0.27%) while the original ppoFEV1 calculation is at 1.78 l ± 0.53 (2.19%). However, if patients require pneumectomy, more complex techniques to determine the ppoFEV1 should be included to stratify risk.

**Supplementary Information:**

The online version contains supplementary material available at 10.1007/s00408-021-00464-4.

## Introduction

Anatomical lung resection is the gold standard for the treatment of early-stage non-small cell lung cancer (NSCLC) [1]. Pulmonary function testing is a cornerstone of the preoperative physiological assessment of patients that is being evaluated for surgical resection. Pulmonary function is often impaired in patients with resectable tumors because of frequent pulmonary comorbidities. Therefore, a precise preoperative assessment, including the measurement of the forced expiratory volume in 1 s (FEV1) and the diffusing capacity of the lung for carbon monoxide (DLCO) is important [2, 3]. The lower threshold values whether patients are suitable for lobectomy or pneumectomy are clearly defined in the guidelines [2–5]. Morbidity rates are substantially increased when lobectomies are performed in patients with FEV1 < 1.5 l, DLCO < 50% or pneumectomies with FEV1 > 2 l, DLCO < 60% [2, 6–8]. The predicted postoperative FEV1 (ppoFEV1) plays a significant role in assessing postoperative lung function [2, 6, 9, 10]. There are various methods available for evaluating the ppoFEV1 such as quantitative CT scans [11], where tumor volume (including the segment or lobe to be resected) is subtracted from total lung volume. Despite those methods there is the long established and simple to use ppoFEV1 calculation method [2, 9]. This approach, which calculates the removed segments in ratio to the remaining, was implemented by Brunelli et al. [2, 9]. The advantage of this method is that pulmonary risk stratification is feasible during multidisciplinary team (MDT) conferences. The method however remains controversial due to its simplicity. Therefore, many different methods were established for a more accurate prediction of the postoperativeFEV1 (postFEV1) [11, 12]. The ppoFEV1 is routinely implemented in our department during MDT. Therefore, we decided to compare and contrast the ppoFEV1 with the postFEV1 one year after surgery. We primarily aimed to estimate the accuracy of the ppoFEV1 for particular baseline characteristics and surgical procedures. Additionally, we wanted to see whether we could identify subpopulations of patients in order to modify ppoFEV1 calculation to improve accuracy compared to the actual postFEV1.

## Material and Methods

All relevant patient data were taken from the electronic hospital information system of our institute. 87 patients voluntarily presented to our out-patient department 1 year after surgery for a routine surgical check-up which included a pulmonary function test. We included all patients who received a postFEV1 in our analysis.

### Calculation of the ppoFEV1

The ppoFEV1 is calculated based on the preoperative FEV1 (preFEV1), the number of functional lung segments resected (y), and the total number of functional segments available at time of resection (z). PpoFEV1 = preFEV1 × 1 − $\left(\frac{y}{z}\right)$ [2, 9, 12]. Unless patients have to undergo a redo operation, the total number of segments for both lungs is 19: 10 in the right lung (3 upper, 2 middle, 5 lower lobe) and 9 in the left lung (5 upper and 4 lower lobe).

### Statistical Analysis

After calculating the ppoFEV1, we compared our results with the postFEV1 1 year after surgery.

We determined accuracy levels and classified the deviation of the ppoFEV1 in relation to the postFEV1. In order to reflect the greatest differences, we defined the accuracy level of ± 2% as most accurate, the accuracy level of ± 10% as moderately accurate, and the accuracy level of ±  > 10% as inaccurate. We performed a subgroup analysis excluding the ppoFEV1 ± 2% from the ppoFEV1 ± 10%, in order to prevent counting ppoFEV1 ± 2% patients twice. These patients were defined as ppoFEV1 ± 10% >  ± 2%. We analyzed baseline characteristics and surgical procedures which eventually resulted in the most accurate- or inaccurate ppoFEV1 with univariate and multivariate analyses.

In cases where univariate analysis showed significant differences, we performed a multinominal regression analysis for further evaluation. Multinomial differences are described by odds ratio (OR) and 95% confidence interval (CI). Categorical variables were analyzed using Pearson’s *χ*^2^ or Fisher’s exact test. Continuous parameters were expressed as mean ± standard deviation (SD) and were analyzed by an unpaired Student *t* test. *p*-value <0.05 was considered statistically significant. Statistical analysis was performed using the SPSS statistical software package (Version 25; IBM, Armonk, NY, USA).

### Customization of the ppoFEV1

Significant multinominal parameters were included in our customizing process. The primary objective of this customization was to determine baseline characteristics or surgical procedures which could be included in the calculation in order to improve the ppoFEV1.

The customizing process is explained in the results part. The formulas calculated by us are the following.

For patients < 60 years, an additional lung segment is added to the calculation ppoFEV1 = preFEV1 × $1-\left(\frac{\text{Lung segments resected} + 1}{\text{Total number of segments}}\right)$. For actively smoking patients with more than 30 packyears one lung segment is subtracted from the calculation ppoFEV1 = PreFEV1 ×  $1-\left(\frac{\text{Lung segments resected} - 1}{\text{Total number of segments}}\right)$.

## Results

A total of 464 patients underwent anatomical pulmonary resections since 2012 at our institution. 87 (18.8%) patients presented voluntarily for a postoperative check-up and a redo assessment of their pulmonary function 1 year after surgery. We classified patients into categories according to how accurately the ppoFEV1 predicted the postFEV1. The ppoFEV1 of 79% patients showed moderate accuracy of ± 10%. 24% of patients were most accurately predicted with a ± 2% deviation. Calculated values exceeded >  ± 10% in 21% of patients. 51 (45%) patients showed a ppoFEV1 ± 10% >  ± 2% accuracy level. The mean preFEV1 of the cohort was 2.34 l (l) ± 0.61 l and the mean postFEV1 was 1.82 l ± 0.47 l, respectively. Calculation of the ppoFEV1 yielded 1.78 l ± 0.53 l (2.19%).

### Baseline Characteristics—Univariate Analysis

The baseline characteristics according to the predictive accuracy level are presented in Tables 1 and 2. We compared patients with the most accurate ppoFEV1 ± 2% with patients with moderate deviation ppoFEV1 ± 10% >  ± 2%. Secondly, we compared the poorest group of ppoFEV1 >  ± 10% with the ppoFEV1 ± 10% group. Age did not differ between ppoFEV1 ± 2% and ppoFEV1 ± 10% >  ± 2%. Patients with ppoFEV1 >  ± 10% on the other hand were significantly younger than ppoFEV1 ± 10% patients (59.7 ± 10.3 years vs 65.1 ± 6.5 years, *p* = 0.01).

**Table 1:** Baseline characteristics ppoFEV1 ± 10% >  ± 2% vs. ppoFEV1 ± 2%

	Total cohort *n* = 87	ppoFEV1 ± 10% ± 2% *n* = 51 (45%)	ppoFEV1 ± 2% *n* = 21 (24%)	Univariate *p*-value	Multivariate *p*-value
Age (years)	63.9 ± 7.7	64.9 ± 6.7	65.1 ± 5.9	0.99	
Female gender *n*. (%)	40 (46)	22 (48.3)	8 (38.1)	0.26	
Preoperative FEV1 (l/s)	2.34 ± 0.61	2.27 ± 0.60	2.33 ± 0.52	0.67	
Preoperative FEV1 in %	84.4 ± 18.2	82.1 ± 15.4	85.8 ± 17.3	0.46	
Postoperative FEV1 (l/s)	1.82 ± 0.47	1.73 ± 0.45	1.84 ± 0.44	0.38	
Preoperative DLCO in %	81.0 ± 19.0	77.3 ± 19.9	87.4 ± 17.7	**0.05**	0.06
Postoperative DLCO in %	78.3 ± 19.8	75.0 ± 18.7	82.5 ± 22.3	0.19	
BMI (kg/m^2^)	25.3 ± 4.2	25.5 ± 4.4	26.1 ± 4.4	0.65	
Smoking					
Active smoking *n*. (%)	65 (74.7)	35 (77.8)	15 (68.4)	0.33	
Smoking in packyears	43.8 ± 24.9	44.9 ± 27.9	33.4 ± 14.1	**0.03**	0.08
Heavy smoker > 30 py *n*. (%)	62 (74.7)	31 (68.9)	15 (71.4)	0.9	
COPD *n*. (%)	31 (35.6)	13 (40.0)	6 (28.6)	0.29	
CVD *n*. (%)	21 (24.1)	18 (26.9)	5 (23.8)	0.47	

Significant values are highlighted in bold

*BMI* body mass index, *COPD* chronic obstructive pulmonary disease, *CVD* cardiovascular disease, *FEV1* forced expiratory volume in 1 s, *ppoFEV1* predicted postoperative FEV1, *DLCO* diffusing capacity for carbon monoxide, *PY* packyears

**Table 2:** Baseline characteristics ppoFEV1 ± 10% vs. ppoFEV1 >  ± 10%

	Total cohort *n* = 87	ppoFEV1 ± 10% *n* = 69 (79%)	ppoFEV1 > ± 10% *n* = 18 (21%)	Univariate *p*-value	Multivariate *p*-value
Age (years)	63.9 ± 7.7	65.1 ± 6.5	59.7 ± 10.3	**0.01**	**0.04**
Female gender *n*. (%)	40 (46)	32 (46.4)	8 (44.4)	0.99	
Preoperative FEV1 (l/s)	2.34 ± 0.61	2.39 ± 0.57	2.60 ± 0.76	**0.05**	0.5
Preoperative FEV1 in %	84.4 ± 18.2	83.8 ± 16.0	86.4 ± 26.0	0.48	
Postoperative FEV1 (l/s)	1.82 ± 0.47	1.83 ± 0.45	1.99 ± 0.55	0.1	
Preoperative DLCO in %	81.0 ± 19.0	80.8 ± 19.7	82.1 ± 17.1	0.94	
Postoperative DLCO in %	78.3 ± 19.8	78.1 ± 20.3	79.1 ± 18.2	0.65	
BMI (kg/m^2^)	25.3 ± 4.2	25.7 ± 4.4	24.8 ± 2.9	0.34	
Smoking					
Active smoking *n*. (%)	74.7	49 (71.0)	18 (100)	**0.005**	**0.01**
Smoking in packyears	43.8 ± 24.9	41.2 ± 24.5	61.4 ± 19.1	**0.002**	**0.008**
Heavy smoker > 30 py *n*. (%)	62 (71.3)	51 (73.9)	18 (100)	**0.001**	**0.004**
COPD *n.* (%)	35.6	25 (36.2)	6 (33.3)	0.52	
CVD *n*. (%)	24.1	18 (26.1)	3 (16.7)	0.38	

Significant values are highlighted in bold

*BMI* body mass index, *COPD* chronic obstructive pulmonary disease, *CVD* cardiovascular disease, *FEV1* forced expiratory volume in 1 s, *ppoFEV1* predicted postoperative FEV1, *DLCO* diffusing capacity for carbon monoxide, *PY* packyears

**Fig. 1:**
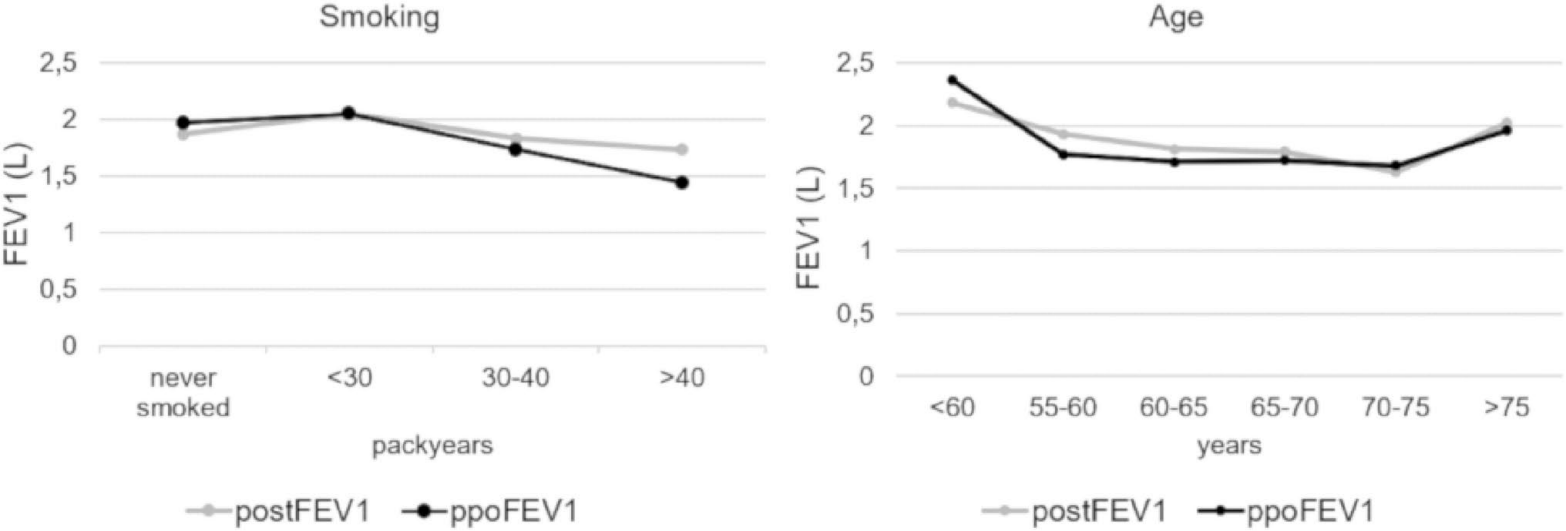
Diagrams showing the comparison of the postoperative FEV1 in liters (gray lines) to the ppoFEV1 in liters (black lines). Comparison of smoking in packyears, Comparison of age in years. *FEV1* forced expiratory volume in 1 s, *postFEV1* postoperative FEV1, *ppoFEV1* predicted postoperative FEV1

The distribution of age in relation of the ppoFEV1 is shown in Fig. 1. The preFEV1 differed significantly between ppoFEV1 >  ± 10% and ppoFEV1 ± 10% (2.61 l ± 0.74 l vs. 2.39 l ± 0.57 l, *p* = 0.05). The preoperative DLCO% (preDLCO%) of patients with ppoFEV1 ± 2% showed a significantly higher value (87.4 ± 17.7) than in ppoFEV1 ± 10 >  ± 2% patients (77.3 ± 19.9) (*p* = 0.05). The postDLCO however did not differ between ± 2% and ± 10 >  ± 2%. 74.7% of patients were active smokers at the time of surgery (active smoking: smoking in 3 months prior to surgery). Active smoking did not differ between ppoFEV1 ± 2% and ppoFEV1 ± 10% >  ± 2%. 100% of ppoFEV1 >  ± 10% patients were active smokers compared to 71.0% of ppoFEV1 ± 10% (*p* = 0.005, Table 2). Preoperative smoking was counted in packyears. The influence of smoking on the postFEV1 is presented in Fig. 1. Patients with ppoFEV1 ± 2% smoked significantly fewer packyears compared to patients with ± 10% >  ± 2% (33.4 ± 14.1 vs. 44.9 ± 27.9, *p* = 0.03). Patients with ppoFEV1 >  ± 10% smoked significantly more than ppoFEV1 ± 10% patients (61.4 ± 19.1 vs. 41.2 ± 24.5 *p* = 0.002). Heavy smoking is defined as > 30 packyears [2, 9]. Heavy smoking did not differ between patients with ppoFEV1 ± 2% and ppoFEV1 ± 10% >  ± 2%. PpoFEV1 >  ± 10% patients were significantly more often heavy smokers than ppoFEV1 ± 10% patients (100% vs. 73.1, *p* = 0.001).

### Baseline Characteristics—Multivariate Analysis

We carried out a multinominal regression analysis for further evaluation of significant results from the univariate analysis (included in Tables 1, 2). In the comparison between the accurate predictive group (ppoFEV1 ± 2%) with moderate ppoFEV1 ± 10% >  ± 2%, we found that neither preDLCO nor the number of packyears were independent factors for a more accurate ppoFEV1 in the multivariate analysis (OR 0.97, CI 0.94–1.02; *p* = 0.07; OR 2.3, CI 0.92–6.12; *p* = 0.07, respectively). However, younger age (OR 1.1, CI 1.01–1.12; *p* = 0.03), active smoking (OR 1.21, CI 0.95–1.62; *p* = 0.01), packyears (OR 4.1, CI 3.6–6.41; *p* = 0.008), and heavy smoking (> 30 packyears) (OR 2.15, CI 0.89–3.12; *p* = 0.004) were independent factors for a more inaccurate ppoFEV1.

### Perioperative Characteristics—Univariate Analysis

**Fig. 2:**
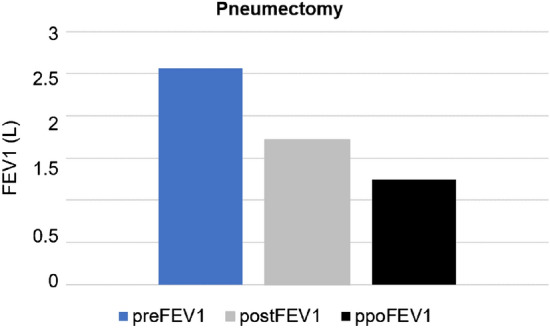
Bar chart showing the comparison of pneumonectomies (*n* = 5) regarding the mean preoperative FEV1 (blue bar), postoperative FEV1 (gray bar), and ppoFEV1 (black bar). *FEV1* forced expiratory volume in 1 s, *preFEV1* preoperative FEV1, *postFEV1* postoperative FEV1, *ppoFEV1* predicted postoperative FEV1

The perioperative characteristics subdivided into different accuracy levels are presented in Tables 3 and 4. Patients with a ppoFEV1 ± 2% underwent a right upper lobe resection significantly more often compared to the ppoFEV1 ± 10% >  ± 2% (42.9% vs. 20.0%, *p* = 0.05). The right lower lobe was resected less often in patients with ppoFEV1 ± 2% (29.9% vs. 9.5%, *p* = 0.04). Patients with ppoFEV1 ± 2% never underwent pneumectomy (0 vs. 3.0%) or bilobectomy (0% vs. 6.0%). The most inaccurate FEV1 >  ± 10% was significantly more frequent in patients undergoing pneumectomy (17.0% vs. 2.9%, *p* = 0.003) (Fig. 2).

**Table 3:** Perioperative characteristics ppoFEV1 ± 10% >  ± 2% vs. ppoFEV1 ± 2%

	Total cohort *n* = 87	ppoFEV1 ± 10% > ± 2% *n* = 51 (45%)	ppoFEV1 ± 2% *n* = 21 (24%)	Univariate *p*-value	Multivariate *p*-value
Left UL-resection *n*. (%)	21 (24.1)	8 (17.8)	8 (38.1)	0.06	
Left LL-resection *n*. (%)	12 (13.8)	7 (15.6)	2 (9.5)	0.7	
Right UL-resection n. (%)	21 (24.1)	9 (20.0)	9 (42.9)	**0.05**	0.17
ML-resection *n*. (%)	6 (6.9)	4 (8.9)	0	0	
Right LL-resection *n.* (%)	18 (20.7)	12 (26.9)	2 (9.5)	**0.04**	0.13
Bi-lobectomy *n*. (%)	4 (4.6)	3 (6.7)	0	0	
Pneumectomy *n*. (%)	5 (5.7)	2 (4.4)	0	0	
Tumor size in cm	2.4 ± 1.7	2.4 ± 1.7	2.4 ± 1.6	0.9	
Adjuvant therapy *n*. (%)	17 (21.8)	8 (17.8)	5 (23.8)	0.25	

Significant values are highlighted in bold

*ppoFEV1* predicted postoperative forced expiratory volume in 1 s, *ML* middle lobe, *LL* lower lobe, *UL* upper lobe

**Table 4:** Perioperative ppoFEV1 ± 10% vs. ppoFEV1 >  ± 10%

	Total cohort *n* = 87	ppoFEV1 ± 10% *n* = 69 (77%)	ppoFEV1 > ± 10% *n* = 18 (21%)	Univariate *p*-value	Multivariate *p*-value
Left UL-resection *n*. (%)	21 (24.1)	16 (23.2)	5 (27.8)	0.76	
Left LL-resection *n*. (%)	12 (13.8)	10 (14.5)	3 (16.7)	0.9	
Right UL-resection *n*. (%)	21 (24.1)	18 (26.1)	3 (16.7)	0.27	
ML-resection *n.* (%)	5 (5.7)	5 (8.7)	0	0	
Right LL-resection *n*. (%)	19 (21.8)	13 (19.0)	5 (27.8)	0.29	
Bi-lobectomy *n*. (%)	4 (4.6)	4 (5.8)	0	0	
Pneumectomy *n*. (%)	5 (5.7)	1 (1.4)	4 (22.2)	**0.004**	**0.02**
Tumor size in cm	2.4 ± 1.7	2.7 ± 1.8	2.1 ± 1.2	0.9	
Adjuvant therapy *n*. (%)	17 (21.8)	13 (19.4)	3 (18.7)	0.35	

Significant values are highlighted in bold

*ppoFEV1* predicted postoperative forced expiratory volume in 1 s, *ML* middle lobe, *LL* lower lobe, *UL* upper lobe

### Perioperative Characteristics—Multivariate Analysis

The multinominal regression of the relevant baseline characteristics is presented in Tables 3 and 4. Both upper and lower lobe resections were no independent factors in the multivariate analysis (OR 0.44, CI 0.12–1.41; *p* = 0.14) (OR 2.28, CI 0.61–6.77; *p* = 0.16). Pneumectomy on the other was an independent factor for ppoFEV1 >  ± 15% in the multivariate analysis (OR 5.65, CI 1.46–23.6; *p* = 0.02) (Fig. 2).

### Customizing the ppoFEV1

According to the results of the multinominal regression analysis, we picked parameters with the greatest impact on the accuracy of the ppoFEV1. Patient age, active smoking status, packyears, and pneumectomy where the parameters with the strongest influence on the accuracy levels of the ppoFEV1. We demonstrated that the calculation of ppoFEV1 was more likely to yield falsely high values in younger patients (Table 2, Fig. 1). We illustrated that active heavy smokers showed lower ppoFEV1 values (Table 2, Fig. 1). Finally, we showed that pneumectomy was an independent factor for an inaccurate measurement of the ppoFEV1 (Table 4, Fig. 2).

To improve the accuracy of the ppoFEV1, we tried to keep the method as simple as possible.

1. We excluded Pneumectomies.
2. We added an additional lung segment to the calculation in patients younger than 60 years.
3. We subtracted one lung segment from the calculation for patients who were active smokers with more than 30 packyears at the time of surgery.
4. When patients were both (2) < 60 years and (3) active heavy smokers we used the original calculation as the modifications canceled each other.

In the following section we give two computational examples with the preFEV1 of two patients explaining our customized ppoFEV1.

Patient undergoing an upper right lobe resection < 60 years:

- ppoFEV1 = preFEV1 x $$1-\left(\frac{\text{Number of lung segments resected} + 1}{\text{Total number of segments}}\right)$$
- preFEV1 = 2.67 l; postFEV1 = 2.04 l; ppoFEV1 2.25 l
- Calculation method: 2.67 l × 1 − $\left(\frac{3}{19}\right)$ = ppoFEV1 = 2.25 l.
- Customized method: 2.67 l × 1 − $\left(\frac{3+1}{19}\right)$ = ppoFEV1 = 2.11 l. Accuracy deviation 6.86%.

Patient undergoing a lower left lobe resection heavy smoker:

- ppoFEV1 = PreFEV1 × $1-\left(\frac{\text{Number of lung segments resected} - 1}{\text{Total number of segments}}\right)$. Actual preFEV1 = 1.77 l; postFEV1 = 1.48 l; ppoFEV1 1.40 l.
- Calculation method: 1.77 × $\left(1-\frac{4}{19}\right)$ = ppoFEV1 = 1.40 l.
- Customized method: 1.77 × $\left(1-\frac{4-1}{19}\right)$ = ppoFEV1 = 1.49 l. Accuracy deviation 4.73%.

**Fig. 3:**
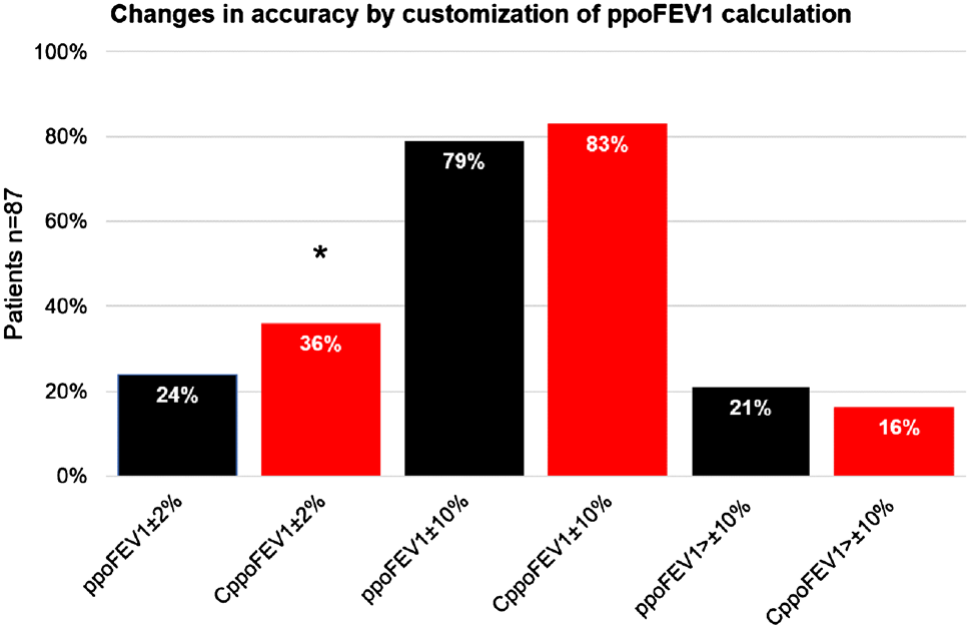
Bar charts showing the comparison of the modified ppoFEV1 (red bars) and the original ppoFEV1 (black bars) subdivided into the accuracy levels ppoFEV1 ± 2%, 10%, and > 10%. Asterisk showing significant values (*p* = 0.01) between original ppoFEV1 ± 2% and customized ppoFEV1. *FEV1* forced expiratory volume in 1 s, *ppoFEV1* predicted postoperative FEV1, *CppoFEV1* customized predicted postoperative FEV1

The mean postFEV1 was 1.823 l ± 0.476 l. By customizing the ppoFEV1 the calculation was more accurate (1.828 l ± 0.479 l) (0.27%) compared to the original ppoFEV1 (1.78 l ± 0.53 l) (2.19%). In addition, customization also led to a redistribution of the accuracy levels we classified (Fig. 3).

## Discussion

### Is the Accuracy of the ppoFEV1 Dependent on Patients’ Characteristics?

The ppoFEV1 is calculated independently of patient’ characteristics [2, 9]. However, factors that influence the accuracy of the ppoFEV1 are being identified. Yokoba et al. found that COPD is an independent factor for an inaccurate ppoFEV1. The authors suggest separating the calculation of patients with and without COPD [13].

Wang et al. demonstrated that COPD decreased the accuracy level of the ppoFEV1 independently [14]. COPD was not an independent factor for an inaccurate or accurate ppoFEV1 in our cohort. In Yokoba's cohort, 28 patients (49.2%) were diagnosed with COPD. In our cohort only 31 (35.6%) were diagnosed with COPD. Emphysema and COPD are substantially underdiagnosed and undertreated in patients under suspicion of lung cancer [15]. Therefore, a higher number of unreported cases can be assumed, especially when we consider the distribution pattern of smoking habits in our cohort. We demonstrated that preoperative smoking habits had a considerable influence on the accuracy levels of the ppoFEV1 in our cohort. Smoking habits in turn directly correlate with the development of COPD [16]. COPD stage could therefore have an impact on ppoFEV1, as stages I and II have a reduced impact on lung function, but patients in stages III and IV show markedly pathological preoperative lung function [6, 7, 10].

We modified the ppoFEV1 for patient smoking habits specifically active smoking status. We further found younger age to be an independent variable for a more inaccurate ppoFEV1 in our cohort. We showed that the ppoFEV1 of patients younger than 55 tended to overestimate compared to the actual postFEV1. This tendency for overestimating the ppoFEV1 decreases in older patients (Fig. 1). To our knowledge this is the first report of this particular inaccuracy. Lung cancer occurs mainly in older people. Most people diagnosed with lung cancer are 65 or older [16, 17]. Consequently, the ppoFEV1 was primarily developed for this age category, so we adjusted the calculation for ppoFEV1 accordingly for younger patients. It could be speculated that younger patients undergo surgery despite decreased pulmonary reserve as they are often otherwise in good health and surgery is their best chance of survival [1, 18]. Elderly patients on the other hand are evaluated more carefully to estimate whether surgery is justifiable in their health condition [19, 20].

### Is the Accuracy of ppoFEV1 Dependent on the Extent of the Resection?

The ppoFEV1 is calculated based on the number of functional lung segments resected in relation to the total number of functional segments overall [2, 9, 12]. We found that the accuracy of the ppoFEV1 depended on the lobes being resected. The ppoFEV1 was more accurate if smaller lobes such as the right upper lobe were resected. The middle lobe was an exception, although only 6 patients in our cohort underwent middle lobe resection. Brunelli et al. and Varela et al. also noted this dependence [9, 21]. The resection of larger lobes such as the lower left lobe resulted in a significantly inaccurate ppoFEV1 [22]. Similar results were published by Yabucchi et al. and Yokoba et al. [22, 23].

The ppoFEV1 for pneumectomy was significantly inaccurate overall in our cohort and showed significant variance (Fig. 2). Brunelli et al. reported similar results regarding extended lung resection, suggesting that measurements of ppoFEV1 for pneumectomies need to be reconsidered [13, 14, 22]. Brunelli et al. showed similar results for extended lung resection, even stating, that measurements of ppoFEV1 for risk stratification for pneumectomies need to be reconsidered [9, 13]. Therefore, we excluded pneumectomy patients for our customized FEV1 (Fig. 2). If pneumectomy is the planned procedure, accurate measurements of the ppoFEV1 are consequently challenging.

### Is the Calculation of the ppoFEV1 Prior to Lobectomy Still Necessary?

According to the guidelines performing lobectomies in patients with a FEV1 < 1.5 l and < 50% DLCO and pneumectomies with a FEV1 < 2 l and DLCO < 60% increases the respiratory morbidity rates substantially [2, 6–8]. Consequently, one could argue that the calculation of ppoFEV1 is not necessary. Many patients are at the lower limit of these values and especially in these borderline patients, the calculation is even more critical [2, 9], because otherwise these patients could be deprived of curative surgery. Ferguson et al. demonstrated in 854 patients that the postFEV1 and ppoFEV1 are strongly associated with long-term survival after pulmonary resection, considerably more so than the preFEV1 [6, 10]. Taking this into account, the calculation of the ppoFEV1 remains particularly important and is therefore still a part of the guidelines [23, 24]. Perhaps our modifications to the ppoFEV1 calculation can help render the evaluation of pulmonary operability more accurate in the future.

## Conclusion

The present study demonstrates that heavy smoking and younger age are independent factors for inaccurate ppoFEV1 calculations. Further, we show that the calculation of the ppoFEV1 is not entirely feasible for younger patients, heavy smokers, or patients undergoing pneumectomy. Therefore, we customized ppoFEV1 calculation taking these measures into account. We excluded patients undergoing pneumectomies, added a lung segment into the calculation for patients < 60 years at the time of surgery and subtracted a lung segment from the calculation for actively smoking patients > 30 packyears at the time of surgery. We were able to provide evidence that these alterations enhance the ppoFEV1. However, if patients require pneumectomy or are in a marginal pulmonary constitution, more complex techniques to determine the ppoFEV1 should be utilized.

## Supplementary Information

Below is the link to the electronic supplementary material.

Supplementary file1 (TIFF 2331 kb)
